# Minipuberty period of children with atopic dermatitis compared to healthy children

**DOI:** 10.55730/1300-0144.5795

**Published:** 2023-11-21

**Authors:** Serkan Bilge KOCA, Hatice EKE GÜNGÖR, Murat CANSEVER

**Affiliations:** 1 Division of Pediatric Endocrinology, Department of Pediatrics, Kayseri City Education and Research Hospital, Health Sciences University, Kayseri, Turkiye; 2 Division of Pediatric Allergy and Immunology, Department of Pediatrics, Kayseri City Education and Research Hospital, Health Sciences University, Kayseri, Turkiye

**Keywords:** Atopic dermatitis, children, hormone, minipuberty

## Abstract

**Background/aim:**

Atopic dermatitis (AD) is an inflammatory, pruritic, noncontagious, chronic relapsing skin disease. Skin barrier abnormalities, excessive T helper 2 activity, and immune dysregulation are held responsible. Androgens have a negative effect on the integrity of the epidermal skin barrier, while estrogen has a positive effect. We aimed to investigate whether hormones make a difference between healthy children and children with AD during minipuberty.

**Materials and methods:**

A total of 96 infants (postnatal 4–13 weeks), 48 diagnosed with AD and 48 controls, were included. Each group consisted of 23 girls (47.9%) and 25 boys (52.1%). Anthropometric examinations and hormone measurements were compared.

**Results:**

The two groups, having similar age, sex, body mass index, and weight-for-length standard deviation scores, were compared. Serum free thyroxine (FT4) levels were found to be lower and insulin-like growth factor binding protein-3 (IGFBP3) levels were found to be higher in children with AD (p < 0.001 and p = 0.038, respectively). In girls with AD, estradiol, FT4, and insulin-like growth factor-1 (IGF-1) levels were found to be lower, but thyroid-stimulating hormone (TSH) levels were found to be higher (p = 0.023, p < 0.001, p = 0.038, and p = 0.034, respectively). In boys with AD, the FT4 level was found to be lower (p = 0.023). Serum FT4 and TSH levels were within normal reference ranges in all comparisons.

**Conclusion:**

Especially in girls with AD, decreased estradiol and IGF-1 levels were observed compared to the controls during minipuberty. In the logistic regression model, decreased levels of serum estradiol, dehydroepiandrosterone sulfate, FT4, and IGF-1, and increased levels of IGFBP3 were associated with an increased likelihood of exhibiting atopic dermatitis.

## 1. Introduction

Activation of the hypothalamic-pituitary-gonadal axis occurs in three stages of life. The first is in fetal life, the second is by the first month of the postnatal period, and the third is by the onset of puberty. The first two periods are temporary and the period observed in the first weeks after birth is called minipuberty [1].

Serum follicle-stimulating hormone (FSH) and luteinizing hormone (LH) levels peak at 1–3 months in male infants and gradually decrease to prepubertal levels around 6–9 months. Serum FSH levels are higher in girls than boys. Although girls’ peak serum FSH levels are similar to those of boys, they may remain elevated until the age of 3–4 years. Girls’ serum LH levels decrease during the same periods as for boys [2,3].

In males, serum testosterone level starts to increase within 1 week following the LH surge and decreases to prepubertal levels until 6 months postnatal. As expected, it is higher in boys than in girls [3]. Serum estradiol levels are high in both sexes at birth. It decreases gradually in the first days of life. Then, only in girls, serum estradiol increases again after the first week [4], and remains high until 6 months postnatal; fluctuating levels are associated with the FSH trend and decrease towards 2 years of age [5].

Atopic dermatitis (AD) is an inflammatory, pruritic, noncontagious, chronic relapsing skin disease, which affects 20% of children and 2%–8% of adults [6]. It is often the first step in the development of other atopic diseases such as allergic rhinoconjunctivitis, asthma, or food allergy [6]. Skin lesions in infants usually first appear at 2–6 months of age [7]. The etiology is complex and not fully understood. Skin barrier abnormalities, excessive T helper 2 (Th2) cell activity, immune dysregulation, and genetic and environmental factors are held responsible [8–10].

Androgens (testosterone, dihydrotestosterone) mostly show immunosuppressive effects. They inhibit T helper 1 (Th1) and Th2 differentiation and interferon-gamma transcription. An increased Th2 response, and increased IL-13 and IgE levels were observed in gonadectomized rats [11–13].

In fetal rats, it has been shown that androgen and progesterone have a negative effect on the integrity of the epidermal skin barrier, while estrogen has a positive effect [14]. On the other hand, estrogen also stimulates mast cell activation and IgE-mediated degranulation [15]. Although it is known that prolactin hormone has a positive effect on skin epithelial cell proliferation and the immune system, no significant difference was found between children aged 0.5–19.5 years with and without AD [16]. However, the minipuberty period was not evaluated separately in that study [16]. While dehydroepiandrosterone sulfate (DHEA-S) increases the Th1 cytokine response, it decreases the Th2 cytokine response [17].

This current study aimed to investigate whether there were differences in androgen and estrogen levels in children with AD, especially in the minipuberty period, and whether there were sex-specific differences.

## 2. Materials and methods

This prospective study was approved by the clinical research ethics committee of Kayseri City Education and Research Hospital. Written informed consent forms were obtained from the legal guardians of all participants. Ethical principles were adhered to and carried out in accordance with the Declaration of Helsinki. This research began in February 2022 and ended in January 2023. Sample size calculation was performed using G*Power version 3.1.9.2 (Kiel University, Kiel, Germany). The sample size was calculated as 45 patients for each group, with 80% power, a 5% significance level, and an effect size value of 0.6. To guard against the possibility of patient dropouts, 48 patients were included in each group; thus, the actual power of the study was determined as 0.80.

### 2.1. Subjects

All individuals participating in this study were evaluated in the pediatric endocrinology and pediatric allergy and immunology outpatient clinics of Kayseri City Hospital in 2022. AD was determined by two pediatric allergists and immunology specialists and evaluated using the Hanifin and Rajka criteria and the Scorad index [18].

Children with isolated AD who were born between 37–42 weeks and whose postnatal age was 4–16 weeks were included in this study. Age and sex-matched healthy infants were included as controls. Those with any systemic disease or allergic immunological disease such as an immunodeficiency were excluded. The children included in the control group were reevaluated at 6 months postnatal to confirm that they did not have findings compatible with AD.

The case and control groups consisted of children who applied to the pediatric endocrinology outpatient clinic. Micropenis, unilateral undescended testis, and premature thelarche were among the reasons for admission. Among the infants who were found to have normal hormonal examinations, those with AD findings were also evaluated in the pediatric allergy outpatient clinic.

### 2.2. Anthropometric measurements

As an anthropometric evaluation, weight, length, body mass index (BMI), weight-for-length values, and age- and sex-specific standard deviation scores (SDSs) of these values were analyzed using an online calculation program (www.childmetrics.org). [19]. The age- and sex-specific reference values were evaluated according to CDC growth cards.

### 2.3. Laboratory measurements

Blood samples for hormone measurements were obtained by skipping a feeding in the early morning after a fasting period of 6–8 hours. The biochemical measurements (glucose, liver, and kidney function test results) of the whole group were normal. Serum FSH, LH, estradiol, testosterone, prolactin, DHEA-S, thyroid stimulating hormone (TSH), free thyroxine (FT4), Anti-Müllerian hormone (AMH), sex hormone binding globulin (SHBG), insulin-like growth factor-1 (IGF-1), and insulin-like growth factor binding protein-3 (IGFBP3) levels were measured using an electrochemiluminescence immunoassay (ECLIA) on the Cobas 8000 e602 analyzer (Roche Diagnostics, Mannheim, Germany).

### 2.4. Statistical analysis

The statistical analysis of the data was done using SPSS version 24.0 (IBM Corp., Armonk, NY, USA). The mean, standard deviation (SD), median, 1st quartile (Q1), and 3rd quartile (Q3) values of numerical variables were calculated. Categorical variables were expressed as numbers and percentages (%). The Shapiro–Wilk test was used to evaluate the normal distribution of the variables. In addition, the variables with kurtosis and skewness values in the range of −0.5 to +1.5 were considered to have a normal distribution. Categorical variables were analyzed using the chi-square test. Student’s t test was used in the comparison of two groups with normally distributed variables, and the Mann–Whitney U test was used if not normally distributed. Clinical and hormonal markers used to predict the presence of AD were analyzed by binary logistic regression analysis. For statistical significance, p < 0.05 was considered significant.

## 3. Results

A total of 96 infants were included in this case–control study. Our sample consisted of 48 children diagnosed with AD and 48 controls. Each group consisted of 23 girls (47.9%) and 25 boys (52.1%). The distribution by sex was similar (χ^2^: p = 1.00). The groups consisted of children at postnatal 4–13 weeks. There was no statistical difference between the groups based on age distribution (p = 0.27).

In terms of food allergy sensitization, 1 child had fish allergy, 2 children had milk allergy, 4 children had egg allergy, and 11 children had multiple food allergies (sesame, hazelnut, and peanut allergy with egg or milk). The Scorad score (mean + standard deviation) of children with AD was 16.4 ± 10.3, with a range from 4 to 50. According to the Scorad score indication of degree, the number of mild cases (score < 25) was 37 (77.1%), the number of moderate cases (score 25–50) was 10 (20.8%), and the number of severe cases (score > 50) was 1 (2.1%). Boys with AD had a higher Scorad score than girls (19.3 ± 12.1 vs 12.8 ± 6.6, p = 0.024).

In the comparison of the two groups with similar age, sex, BMI, and weight-for-length SDS, serum FT4 levels were found to be lower and serum IGFBP3 levels were found to be higher in children with AD (p < 0.001 and p = 0.038, respectively). The serum FT4 levels were within normal reference ranges. The comparison of hormone results during minipuberty between children with AD and the control group are shown in Table 1.

The analyses were repeated by dividing the AD and control group into two subgroups of girls and boys. When comparing girls with AD with girls in the control group, there was no statistical difference between age and weight-for-length SDSs (p = 0.082 and p = 0.759, respectively). In girls with AD, serum estradiol, FT4, and IGF-1 levels were found to be lower, but serum TSH levels were found to be higher (p = 0.023, p < 0.001, p = 0.038, and p = 0.034, respectively). Serum TSH and FT4 levels were within normal reference ranges. The comparison of hormone results during minipuberty between girls with AD and girls in the control group are shown in Table 2.

When comparing boys with AD with boys in the control group, there was no statistical difference between age and weight-for-length SDSs (p = 1.00 and p = 0.067, respectively). In boys with AD, serum FT4 levels were found to be lower (p = 0.023), but within normal reference ranges. The comparison of hormone results during minipuberty between boys with AD and boys in the control group are shown in Table 3.

A logistic regression model was performed to determine the effects of clinical (age, sex, weight SDS, length SDS, and weight-for-length SDS), and hormonal (serum FSH, LH, estradiol, testosterone, prolactin, DHEA-S, TSH, FT4, IGF-1, IGFBP3, SHBG, AMH) markers on the cases with AD. The logistic regression model was statistically significant, χ^2^(17) = 63.918, p < 0.001. The model explained 65.8% (Nagelkerke R2) of the variance in AD and correctly classified 86.2% of cases. That is, of all cases predicted as AD, 82.7% were correctly predicted (the positive predictive value), and of all cases predicted as not AD, 90.5% were correctly predicted (the negative predictive value).

Decreasing levels of serum estradiol, DHEA-S, FT4, and IGF-1, and increasing levels of IGFBP3 were associated with an increased likelihood of exhibiting AD. Univariate binary logistic regression analysis results of factors that predict AD are shown in Table 4.

## 4. Discussion

The minipuberty period is an area open to research that has not yet been fully elucidated from an endocrine point of view. To the best of our knowledge, this is the first study to evaluate the minipuberty period in children with AD and compare it with a control group. In this case–control study, two groups in the minipuberty period with similar age, sex, and weight-for-length SDS characteristics were compared. Sex-specific hormonal changes have been observed during minipuberty in children with AD. Especially in girls with AD, decreased estradiol and IGF-1 levels were observed during minipuberty compared to controls. There was no statistically significant difference between the groups in terms of serum testosterone, DHEA-S, prolactin, AMH, and SHBG levels. In the logistic regression model, decreasing levels of serum estradiol, DHEA-S, FT4, and IGF-1, and increasing levels of IGFBP3 were associated with an increased likelihood of exhibiting AD during minipuberty.

One study showed that GH and IGF-I induce IgE and IgG4 production, via an IL-4 and IL-13 independent mechanism [20]. Another study reported that insulin and IGF-1 support mast cell survival through activation of the phosphatidylinositol-3-kinase pathway [21]. Moreover, IGFBP-3 has been shown to inhibit airway inflammation and hyperresponsiveness through an IGF-independent mechanism that includes activation of IGFBP-3 receptor signaling and cross-talk with NF-κB signaling [22]. In our study, IGFBP3 levels were found to be higher in children with AD than in the control group. When the subgroups were analyzed according to sex, while IGF-1 levels were lower in girls with AD, no significant difference was found in the IGFBP3 levels of the two groups. Moreover, no significant difference was found between IGF-1 and IGFBP3 levels in boys. In addition, serum estradiol levels were found to be lower in girls with AD. Although a cause–effect relationship could not be determined with these results, it shows the minipubertal period in girls was more active than that in boys.

In a study using older patients, about half of the female AD patients experienced worsening of their symptoms during the premenstrual period due to the dual effects of estrogen and progesterone on Th2 activity. It is thought that the skin barrier can be disrupted, especially just before menstruation, which affects the minimum estrogen/progesterone ratio [23]. The skin is a target tissue on which the effect of thyroid hormone is well known. Thyroid hormones have effects such as fetal epidermal differentiation, barrier formation, hair growth, wound healing, keratinocyte proliferation, and keratin gene expression [24,25].

One study demonstrated that the prevalence of thyroid autoimmunity is higher in children affected by AD, and even higher in IgE-mediated AD than in non-IgE-mediated [26]. In another study, when children with allergic disease were evaluated compared to controls, the prevalence of subclinical hypothyroidism was found to be statistically higher in atopic individuals and, additionally, in those with non-atopic allergic diseases [27]. Although there are some known changes in autoimmune thyroid diseases and thyroid function tests in advanced age in individuals with AD, we have limited information about the differences in early infancy or minipuberty.

In our study, although the serum FT4 level was within the normal reference ranges (euthyroid), it was found to be lower in children with AD compared to the controls. Moreover, in the boys and girls subgroup comparisons, serum FT4 levels were found to be lower in the AD group, even though they were euthyroid. Although the serum TSH levels of the AD girls were within the normal reference ranges, their serum TSH levels were found to be statistically higher than the control.

Furthermore, it has been shown in a study that IGF-1 levels have positive effects on the suppression of inflammation, cell proliferation, and wound healing by directing T regulatory cells in cases with allergic contact dermatitis, and a similar effect can even be obtained with topical and systemic IGF-1 given for therapeutic purposes [28].

One of the limitations of our study was that our examinations in the minipuberty period were cross-sectional and not longitudinal. Secondly, the detected hormone changes did not reflect a cause–effect relationship. In other words, we cannot answer the questions of whether these hormone changes occur in children with AD, or whether these hormone changes can be a risk factor for AD. Third, our sample size was relatively small.

## 5. Conclusion

This is the first study to examine the relationship between AD and minipuberty. In the logistic regression model, decreasing levels of serum estradiol, DHEA-S, FT4, and IGF-1, and increasing levels of IGFBP3 were associated with an increased likelihood of exhibiting AD. Although our study revealed cross-sectional results, we hope that it can also guide longitudinal studies that include more children.

## Figures and Tables

**Table 1:** Comparison of hormone results during minipuberty between children with AD and the control group.

	AD group		Control group		p
	Mean ± SD	Median (Q1–Q3)	Mean ± SD	Median (Q1–Q3)	
Age (weeks)	8.5 ± 2.5	9 (6–10.8)	7.9 ± 2.6	7 (6–10)	0.27
Weight (kg)	5.7 ± 0.87	5.6 (5–6.4)	5.2 ± 0.84	4.9 (4.7–5.7)	**0.003**
Length (cm)	57.6 ± 3.3	58 (55–60)	54.9 ± 3	55 (53–56)	**<0.001**
BMI (kg/m2)	17.1 ± 1.7	17 (15.9–18.3)	17.1 ± 1.65	16.9 (16–17.7)	0.797
Weight SDS	0.68 ± 1.03	0.67 (−0.03 to 1.52)	0.28 ± 0.83	0.37 (−0.45 to 0.87)	**0.039**
Length SDS	−0.2 ± 0.97	−0.16 (−0.9 to 0.66)	−0.87 ± 0.89	−1.03 (−1.55 to −0.28)	**0.001**
Weight-for-length SDS	0.87 ± 1.11	0.95 (0.26–1.46)	1.23 ± 0.93	1.32 (0.56–2.08)	0.094
FSH (U/L)	3.57 ± 3.54	2.3 (1.76–4.34)	4.3 ± 6.1	2.24 (1.27–3.79)	0.639^*^
LH (U/L)	2.17 ± 2.26	1.55 (0.2–3.8)	2.69 ± 2.53	2.35 (0.25–4.6)	0.285
Estradiol (ng/L)	6.4 ± 3.5	5 (5–5)	7.7 ± 4.5	5 (5–10.1)	0.086^*^
Testosterone (μg/L)	87 ± 100.3	53 (2.5–170.3)	81.9 ± 98.6	40 (8.5–139)	0.72^*^
Prolactin (μg/L)	34.2 ± 26.3	30 (16.3–43.3)	36.6 ± 19.1	31.5 (22–46)	0.177^*^
DHEA-S (μg/dL)	47.4 ± 29.9	39.5 (22.3–68.5)	67.9 ± 55.3	53.5 (27–82.8)	0.12^*^
TSH (mU/L)	3.08 ± 1.63	2.89 (1.8–4.09)	2.77 ± 1.89	2.62 (1.14–4.45)	0.383
FT4 (ng/L)	14.2 ± 1.7	14.1 (12.9–15.2)	16.3 ± 2.7	16.2 (14.5–17.8)	**<0.001**
IGF-1 (ng/mL)	55.9 ± 20.2	52 (43–71)	61.7 ± 19.5	59.5 (48–74.8)	0.158
IGFBP-3 (ng/mL)	2292.9 ± 722	2215 (1910–2470)	2046.4 ± 457	2040 (1830–2340)	**0.038** ^*^
SHBG (nmol/L)	113.8 ± 39.7	108 (83–142)	110.1 ± 32.2	103 (84.3–124)	0.617
AMH (μg/L)	17.7 ± 17	23 (1.99–23)	15.3 ± 13.7	23 (1.68–23)	0.444

Data with a normal distribution were examined using Student’s t test. Variables without a normal distribution were evaluated using the Mann–Whitney U test, as indicated by the ^*^ symbol. Statistically significant p-values are shown in bold.

Abbreviations: BMI: body mass index, SDS: standard deviation score, FSH: follicle-stimulating hormone, LH: luteinizing hormone, DHEA-S: dehydroepiandrosterone sulfate, TSH: thyroid-stimulating hormone, FT4: free thyroxine, IGF-1: insulin-like growth factor-1, IGFBP-3: insulin-like growth factor binding protein-3, SHBG: sex hormone binding globulin, AMH: anti-Müllerian hormone.

**Table 2:** Comparison of hormone results during minipuberty between girls with AD and girls in the control group.

	Girls with AD		Control girls		p
	Mean ± SD	Median (Q1–Q3)	Mean ± SD	Median (Q1–Q3)	
Age (weeks)	9 ± 2.3	9 (8–11)	7.7 ± 2.4	7 (6–10)	0.082
Weight (kg)	5.7 ± 0.67	5.6 (5.1–6.4)	4.9 ± 0.66	4.9 (4.5–5.2)	**<0.001**
Length (cm)	57.3 ± 2.7	56 (55–59)	54.1 ± 2.99	54 (52–55)	**0.001**
BMI (kg/m2)	17.4 ± 1.3	17.1 (16.3–18.3)	16.6±1.3	16.6 (15.6–17.4)	0.049
Weight SDS	0.87 ± 0.91	0.68 (0.34–1.57)	0.25 ± 0.95	0.23 (–0.52 to 0.97)	**0.03**
Length SDS	−0.2 ± 0.8	−0.15 (−0.9 to 0.54)	−0.83 ± 1.02	−0.83 (−1.75 to −0.06)	0.024
Weight-for-length SDS	1.12 ± 0.81	1.13 (0.52–1.69)	1.2 ± 0.93	1.28 (0.55–2.09)	0.759
FSH (U/L)	5.43 ± 4.32	3.83 (2.48–7.22)	7.1 ± 7.91	3.74 (2.07–10.4)	0.869^*^
LH (U/L)	0.34 ± 0.38	0.2 (0.1–0.4)	1.2 ± 2.42	0.4 (0.1–0.9)	0.353^*^
Estradiol (ng/L)	6.8 ± 4.4	5 (5–6.4)	9.4 ± 5.1	7.9 (5–12.4)	**0.023** ^*^
Testosterone (μg/L)	7.3 ± 9.7	2.5 (2.5–12.7)	10.7 ± 10.1	8.5 (2.5–15.3)	0.056^*^
Prolactin (μg/L)	35.7 ± 32.1	31 (15–41)	37.4 ± 14.1	32 (26–49)	0.223^*^
DHEA-S (μg/dL)	41.1 ± 33.1	27 (18–56)	56.2 ± 44.5	39 (26–65)	0.099^*^
TSH (mU/L)	3.57 ± 2	2.93 (2.2–4.89)	2.3 ± 1.9	1.9 (0.58–4.37)	**0.034**
FT4 (ng/L)	13.8 ± 1.4	13.7 (12.6–15)	16.2 ± 2.4	16.4 (14.5–18.2)	**<0.001**
IGF-1 (ng/mL)	53 ± 17.9	48 (42–65.5)	64.8 ± 19.1	64 (48–75)	**0.038**
IGFBP-3 (ng/mL)	2236.1 ± 353	2220 (1910–2470)	2142.7 ± 442	2045 (1832–2367)	0.203^*^
SHBG (nmol/L)	113.5 ± 35.1	109.5 (86–140.5)	106.8 ± 33	102 (84–125)	0.515
AMH (μg/L)	1.99 ± 1.58	1.79 (0.42–3.37)	3.02 ± 4.86	1.6 (0.57–3.2)	0.847^*^

Data with a normal distribution were examined using Student’s t test. Variables without a normal distribution were evaluated using the Mann–Whitney U test, as indicated by the ^*^ symbol. Statistically significant p-values are shown in bold.

Abbreviations: BMI: body mass index, SDS: standard deviation score, FSH: follicle-stimulating hormone, LH: luteinizing hormone, DHEA-S: dehydroepiandrosterone sulfate, TSH: thyroid-stimulating hormone, FT4: free thyroxine, IGF-1: insulin-like growth factor-1, IGFBP-3: insulin-like growth factor binding protein-3, SHBG: sex hormone binding globulin, AMH: anti-Müllerian hormone.

**Table 3:** Comparison of hormone results during minipuberty between boys with AD and boys in the control group.

	Boys with AD		Control boys		p
	Mean ± SD	Median (Q1–Q3)	Mean ± SD	Median (Q1–Q3)	
Age (weeks)	8 ± 2.7	8 (5.5–10.5)	8 ± 2.9	8 (6–10)	1.00
Weight (kg)	5.7 ± 1.03	5.5 (5–6.3)	5.4 ± 0.92	5.2 (4.7–6.2)	0.344
Length (cm)	57.9 ± 3.9	58 (55–60)	55.6 ± 2.9	55 (53–57.5)	**0.023**
BMI (kg/m2)	16.9 ± 2	16.5 (15.3–18.3)	17.5 ± 1.9	16.9 (16.2–18.5)	0.324
Weight SDS	0.51 ± 1.12	0.56 (−0.4 to 1.11)	0.31 ± 0.72	0.38 (−0.41 to 0.83)	0.454
Length SDS	−0.2 ± 1.13	−0.16 (−1.33 to 0.9)	−0.91 ± 0.76	−1.18 (−1.44 to −0.49)	**0.012**
Weight-for-length SDS	0.65 ± 1.3	0.87 (−0.16 to 1.25)	1.25 ± 0.95	1.5 (0.49–2.11)	0.067
FSH (U/L)	1.85 ± 0.96	1.81 (1.01–2.27)	1.73 ± 1.12	1.41 (0.91–2.41)	0.473^*^
LH (U/L)	3.84 ± 1.93	3.8 (2.25–4.95)	4.07 ± 1.76	3.6 (2.95–5.35)	0.665
Estradiol (ng/L)	6 ± 2.6	5 (5–5)	6.2 ± 3.3	5 (5–5)	1.00^*^
Testosterone (μg/L)	160 ± 89	141 (89–200)	147.5 ± 98.1	127 (84–190)	0.607^*^
Prolactin (μg/L)	32.8 ± 20.1	26 (17.5–45.5)	35.8 ± 23	28 (18–43.5)	0.626
DHEA-S (μg/dL)	53.2 ± 25.9	47 (34–77)	78.6 ± 62.7	66 (28–83)	0.248^*^
TSH (mU/L)	2.63 ± 1.1	2.89 (1.8–3.28)	3.19 ± 1.77	3.1 (1.8–4.6)	0.182
FT4 (ng/L)	14.6 ± 2	14.3 (13–15.8)	16.3 ± 3	16.1 (13.5–17.7)	**0.023**
IGF-1 (ng/mL)	58.6 ± 22	55 (44–79.5)	58.9 ± 19.8	54 (44–74)	0.952
IGFBP-3 (ng/mL)	2345.2 ± 949	2210 (1775–2555)	1961.6 ± 462	1990 (1740–2335)	0.095^*^
SHBG (nmol/L)	114.1 ± 44.1	102 (75–152.5)	113.1 ± 31.8	109 (88.5–126)	0.927
AMH (μg/L)	31.6 ± 11.1	23 (23–46)	26.6 ± 8.3	23 (23–23)	0.079

Data with a normal distribution were examined using Student’s t test. Variables without a normal distribution were evaluated using the Mann–Whitney U test, as indicated by the ^*^ symbol. Statistically significant p-values are shown in bold.

Abbreviations: BMI: body mass index, SDS: standard deviation score, FSH: follicle-stimulating hormone, LH: luteinizing hormone, DHEA-S: dehydroepiandrosterone sulfate, TSH: thyroid-stimulating hormone, FT4: free thyroxine, IGF-1: insulin-like growth factor-1, IGFBP-3: insulin-like growth factor binding protein-3, SHBG: sex hormone binding globulin, AMH: anti-Müllerian hormone.

**Table 4:** Univariate binary logistic regression analysis of factors that predict AD presence.

Predicting factors	B	p	Odds ratio	95% confidence interval
Age	−0.446	0.051	0.640	0.409–1.003
Sex	−0.707	0.718	0.493	0.011–22.949
Weight SDS	3.989	0.062	53.993	0.822–3547.592
Length SDS	−2.304	0.235	0.100	0.002–4.469
Weight-for-length SDS	−3.209	0.075	0.040	0.001–1.385
FSH	−0.014	0.896	0.986	0.804–1.210
LH	−0.455	0.076	0.634	0.384–1.048
Estradiol	−0.199	**0.041**	0.819	0.676–0.992
Testosterone	0.006	0.200	1.006	0.997–1.016
Prolactin	0.007	0.666	1.007	0.977–1.037
DHEA-S	−0.027	**0.028**	0.973	0.950–0.997
TSH	0.035	0.866	1.036	0.686–1.564
FT4	−0.772	**<0.001**	0.462	0.300–0.713
IGF-1	−0.084	**0.006**	0.920	0.866–0.976
IGFBP-3	0.004	**0.005**	1.004	1.001–1.007
SHBG	0.007	0.550	1.007	0.983–1.032
AMH	0.020	0.647	1.021	0.935–1.113

BMI: body mass index, SDS: standard deviation score, FSH: follicle-stimulating hormone, LH: luteinizing hormone, DHEA-S: dehydroepiandrosterone sulfate, TSH: thyroid-stimulating hormone, FT4: free thyroxine, IGF-1: insulin-like growth factor-1, IGFBP-3: insulin-like growth factor binding protein-3, SHBG: sex hormone binding globulin, AMH: anti-Müllerian hormone. Statistically significant p-values are shown in bold.
